# Analyzing the association between social vulnerability indexes and surgically underserved areas in the Inland Empire

**DOI:** 10.1016/j.sopen.2023.12.008

**Published:** 2024-01-05

**Authors:** Brandon J. Shin, M. Daniel Wongworawat, Marti F. Baum

**Affiliations:** Loma Linda University School of Medicine, Loma Linda, CA, USA

**Keywords:** Social vulnerability, Surgery, Disparity, Income, Access

## Abstract

**Background:**

The United States lacks equitable surgical access, prompting us to investigate whether there is an inverse relationship between Social Vulnerability Indices and the number of surgeons in a census tract, using the Inland Empire as a model.

**Methods:**

The Centers for Disease Control's (CDC) SVI 2018 database, composed of 823 census tracts, was compared against demographics of 1008 surgeons, from the American Medical Association's (AMA) 2018 Physician Masterfile. Analysis was performed via Spearman's bivariate and multiple regression.

**Results:**

An inverse relationship exists between surgeon number and overall social vulnerability (ρ = −0.266 [95 % CI −0.330 to −0.199], p < .001), and between surgeon number and each category of social vulnerability: Socioeconomic (ρ = −0.345 [95 % CI −0.0405 to −0.281], p < .001), Household Composition and Disability (ρ = −0.121 [95 % CI −0.190 to −0.051], p < .001), Minority Status and Language (ρ = −0.0317 [95 % CI −0.379 to −0.252], p < .001), and Housing Type and Transportation (ρ = −0.093 [95 % CI −0.153 to −0.023], p = .005). Multiple regression analysis revealed that the following were associated with a higher number of surgeons: higher “Per Capita Income” (B = 0.000151 [95 % CI 0.000079 to 0.000223], t(820) = 4.104, p < .001), larger Daytime Population (B = 0.000143 [95 % CI 0.000072 to 0.000214]; t(820) = 3.956, p < .001), larger Total Population (B = −0.013 [95 % CI −0.022 to −0.003]; t(820) = −2.672, p = .008), and smaller number of Persons aged 17 and younger (B = −0.005 [95 % CI −0.008 to −0.001]; t(820) = −2.794, p = .005).

**Conclusions:**

This study concludes that social vulnerability is predictive of, and significantly linked to, differences in surgical access and continues to advocate for research into understanding the surgeon's role in both individual and population health.

**Key message:**

Our work demonstrates that the number of surgeons in a census tract is inversely proportional to the census tract's overall Social Vulnerability Indices. Thus, this research can serve to educate the public, physicians, and other healthcare providers about the importance of incorporating social determinants of health into the construction of healthcare policy and practice, as well as the importance of continued funding for local and national social service programs as a means to alleviate specific health inequities, such as language and transportation.

## Highlights

• Most surgical centers are based in large metropolitan areas.
• There is an inverse relationship between surgeons and overall social vulnerability.
• A significant number of census tracts in the Inland Empire do not have any surgeons
• Incorporating social health into healthcare policy increases access to surgical care.

## Introduction

Globally, people with higher incomes are much more likely to see a physician than those with a lower income, with the United States demonstrating some of the largest gaps in healthcare access [1]. This unequal distribution of access to medicine in the United States is heavily influenced by geographic segregation of individuals based on socioeconomic factors deeply tied to health and longevity, such as race and income [2]. Furthermore, predominantly poorer communities are typically composed of minority groups, and tend to face problems that their wealthier counterparts do not, such as generational poverty, food deserts, unstable housing costs, and subpar access to housing and social services (e.g., medical care) [2]. Thus, the unequal distribution of medical care across the geography of the United States has led to the propagation of medically underserved areas (MUAs), populations deemed by the Health Resources and Services Administration as having high poverty, high infant mortality, a large elderly population, or not enough primary care providers [3]. However, although significant research has investigated the relationship between socioeconomic status and access to primary care physicians, there is a lack of equivalent research into access to surgeons [[4], [5], [6]].

Like other forms of medical care, most surgical centers are typically based in large metropolitan areas, making it difficult for those outside of wealthier, high-density cities to access quality surgical interventions [7]. This has led to the development of surgically underserved areas or SUAs. In 2006, one-third of the counties in the United States lacked any surgeons [8], while others struggled balancing the need of the patient population with surgeon supply [7]. This surgical shortage is further pronounced when analyzing the distribution of surgical subspecialties. For instance, the American Association for Thoracic Surgery anticipates a critical lack of cardiothoracic surgeons by 2035, due to a failure of a proportional increase in residents alongside an increasing surgical workload [9]. Furthermore, it is likely that those without access to general surgery will not have access to surgical subspecialties, due to the increased requirements for equipment and resources [7]. Thus, the continued decrease in the number of general surgeons, specialist surgeons, and the non-interchangeability of surgeons between specialties, indicates a troubling lack of surgical access for many.

In response to these community-based differences in variables such as access to food, housing, and medical care, the Centers for Disease Control (CDC) developed Social Vulnerability Indices (SVIs). SVIs are a series of characteristics used to describe and quantify the risk factors experienced by different census tracts across the United States [10], and are grouped into four major categories: Socioeconomic, Household Composition and Disability, Minority Status and Language, and Housing Type and Transportation, with other sub-characteristics included within each category.

The Inland Empire (IE), an informally designated geographic area in Southern California centering around San Bernardino and Riverside counties, exhibits varied stratifications across nearly every SVI, including transportation access, education level, exposure to pollution, and income [10,11]. For instance, Loma Linda University's 2016 Community Health Assessment indicates that San Bernardino County displays poorer levels of prenatal care and food access, and higher levels of teenage pregnancy compared to, not only to its neighboring Riverside County, but also the rest of California. The rates for “all-cause mortality” followed a similar pattern, with San Bernardino exhibiting significantly higher rates than other counties. Furthermore, women and ethnic minorities suffered the highest rates of hospitalizations and severe illnesses across all counties [12]. As a result, the IE, with its juxtaposition of wealthier cities, such as Loma Linda and Chino Hills, alongside their less socioeconomically advantaged counterparts, such as San Bernardino and Perris, serves as an insightful model for the larger patterns of healthcare access disparity found in cities and counties across the larger United States [11].

Informed by these realities, we seek to answer the question: Is there a statistically significant inverse relationship between SVIs and the number of surgeons in a census tract? We hypothesize that the number of surgeons in a census tract will be inversely proportional to that tracts' SVIs (i.e., the number of surgeons will decrease as a census tract's risk factors increase). We also look to elucidate which of the four SVI categories is most related to the number of surgeons, as well as which census tract sub-characteristic is most associated with the number of surgeons.

## Methods

In our investigation into the relationship between various SVIs and the number of surgeons per census tract, we utilized the Inland Empire, a small, loosely defined geographic area in Southern California, as a sample for the larger United States, due to two reasons. The first being that the IE serves as a readily available set of local information for which we can easily and effectively access the relevant data for our investigation (e.g., SVIs, numbers of surgeons). Second, and perhaps more importantly, the IE serves as an insightful demonstration of how the unequal burden of health upon marginalized populations affects health outcomes. For instance, the various census tracts, counties, and zip codes within the IE exhibit large variations in all four categories of social vulnerability: socioeconomic status, household composition and disability, minority status and language, and housing type and transportation. Thus, the IE reflects the same patterns of sociologic inequity seen across the larger United States, allowing us to further investigate the systems that continue to perpetuate health, and specifically surgical, disparity within the United States of America [10,12,13].

**Fig. 1:**
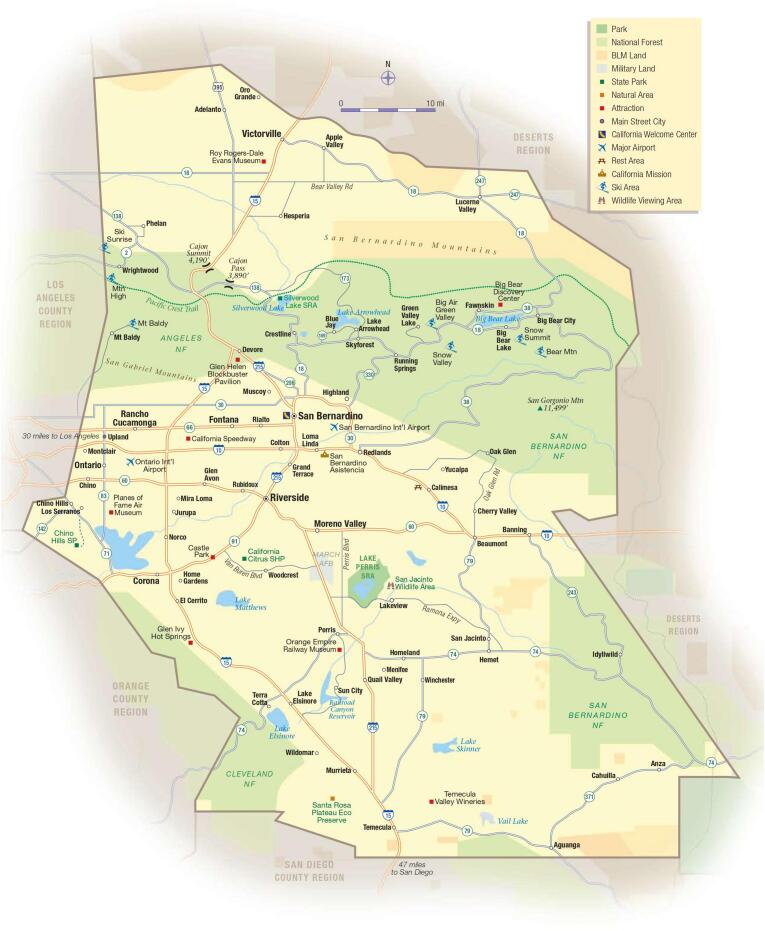
Map of the Inland Empire [29]. Accessed from popumaps.blogspot.com [site removed].

The term ‘Inland Empire’ is, perhaps surprisingly, fraught with contention regarding its boundaries. Unlike other legally delineated areas, such as counties and cities, “‘Inland Empire’ is a made-up term and the areas to which it applies have been argued over for a century” [14]. Thus, as local reporters, politicians, and residents continue to debate the boundaries of the IE, a continuous theme emerges, where “almost everyone who answered believes that they, personally, live in the IE, but they weren't so sure about anyone else” [15]. However, due to the widespread prevalence of the usage of the term, as well as the emotional connection many residents hold towards what is affectionately called, “the IE,” this study applied a more conservative definition, incorporating only the areas most often agreed upon - namely, the areas classified in this study as part of the IE are: San Bernardino and Riverside counties [16] (Fig. 1).

In order to investigate this question of whether the IE has a lower number of surgeons in locations with higher Social Vulnerability Indices (SVIs), compared to locations with lower SVIs, two major resources were utilized: The first was the CDC SVI indices database for California. The CDC provides a series of calculated SVIs which quantify social vulnerability within counties and communities in a number, based on statistics such as the median number of people in a household, the number of languages other than English spoken, the risk of becoming unhoused, a household's median income, etc. This information is then sorted by census tract, providing a numerical insight into a particular geographic region's social vulnerability, with higher SVI numbers equating to higher vulnerability. The most updated set of SVIs for San Bernardino County, tabulated in 2018 and composed of 823 census tracts, were utilized in this project, to ensure that information was as recent and accurate as possible. The second resource was the American Medical Association's Physician Masterfile, purchasable from Medical Marketing Services [17]. This provided information on such as physician specialty, residency location, residency specialty, years of practice, and importantly, census tract of residence. From this database, we selected all currently practicing surgeons (e.g., general, cardiothoracic, plastics) who resided in San Bernardino County, excluding those who were deceased or no longer practicing, as these individuals are not impacting a community's access to surgical care; this yielded 1008 surgeons. The Masterfile data from 2018 was utilized, to ensure that the inter-census tract comparisons and relationships were not confounded by differences in date.

Utilizing the physician list in the Masterfile, the number of surgeons in each census tract was totaled and compared to the various SVIs in each census tract (e.g., 30 surgeons in census tract 1, 3 surgeons in census tract 2, etc.). Thus, these two resources were utilized in order to compare the number of surgeons per census tract alongside the various socioeconomic vulnerabilities of those same communities, focusing primarily on determining whether or not there lies a relationship between a census tract's social vulnerability and the number of surgeons within that census tract. This allowed for a careful examination of how socioeconomic status, household composition, minority status, and housing type affects one's ability to readily access surgery in the IE. Census tracts not listed in the physician Masterfile were assumed to have zero surgeons.

Data analysis was conducted in two forms: First, a Spearman's bivariate analysis was utilized to elucidate the relationship between the various SVIs and the number of surgeons per each particular census tract. The SVIs utilized in this analysis were imported from the CDC's list of measured SVIs for each census tract. This regression was then converted to a p-value to determine statistical significance, with p < .01 signifying significance. Following the Spearman's analysis, a multiple regression analysis was performed via SPSS. This multiple regression plotted the same SVIs utilized in the Spearman's model, with surgeon number serving as the independent variable, to determine which of the previously investigated census tract characteristics was most associated with surgeon number. Utilizing the same SVIs across both analytical methodologies allowed us to investigate relationship in a consistent manner.

## Results

Spearman's Bivariate analysis revealed a statistically significant inverse relationship between the number of surgeons in a census tract and the overall social vulnerability index of that census tract (ρ = −0.266 [95 % CI −0.330 to −0.199], p < .001; Table 1). In addition, analysis demonstrated a statistically significant inverse relationship for all four subcategories of vulnerability: Socioeconomic (ρ = −0.345 [95 % CI −0.0405 to −0.281], p < .001), Household Composition and Disability (ρ = −0.121 [95 % CI −0.190 to −0.051], p < .001), Minority Status and Language (ρ = −0.0317 [95 % CI −0.379 to −0.252], p < .001), and Housing Type and Transportation (ρ = −0.093 [95 % CI −0.153 to −0.023], p = .005). “Socioeconomic” exhibited the strongest inverse relationship amongst the four major categories.

**Table 1:** Table detailing the Spearman rho values for the four major categories of social vulnerability, as well as the sum of the categories, when compared with number of surgeons per census tract.

Spearman relationships		
Social vulnerability index	Spearman's ρ (95 % CI)	p
Socioeconomic	−0.345 (−0.0405, −0.281)	p < .001
Household composition	−0.121 (−0.190, −0.051)	p < .001
Minority status/language	−0.0317 (−0.379, −0.252)	p < .001
Housing type/transportation	−0.093 (−0.153, −0.023)	p = .007
Sum of indices	−0.266 (−0.330, −0.199)	p < .001

Multiple regression analysis revealed that, in a particular census tract, the following were associated with a higher number of surgeons: higher “Per Capita Income” (B = 0.000151 [95 % CI 0.000079 to 0.000223], t(820) = 4.104, p < .001), larger Daytime Population (B = 0.000143 [95 % CI 0.000072 to 0.000214]; t(820) = 3.956, p < .001), larger Total Population (B = −0.013 [95 % CI −0.022 to −0.003]; t(820) = −2.672, p = .008), and smaller number of Persons aged 17 and younger (B = −0.005 [95 % CI −0.008 to −0.001]; t(820) = −2.794, p = .005) (Table 2, Table 3).

**Table 2:** Table displaying the coefficient values for the multiple regression analysis, with surgeon number serving as the dependent variable. Further values can be found in Table 3.

Multiple regression			
SVI subcategory	B (95 % CI)	p (significance)	Beta
Total population	−0.013 (−0.022, −0.003)	p = .008	−0.717
Per capita income	0.000151 (−0.000079, 0.000223)	p < .001	0.419
Persons aged 17 and younger	−0.005 (−0.008, −0.001)	p = .005	−1.047

**Table 3:** Table detailing specific multiple regression values for various relationships with number of surgeons per census tract.

Coefficients					
	B (95 % CI)	Std. error	t	p (significance)	Beta
Tract area in square miles	4.137E−5 (−0.001, 0.001)	<0.001	0.085	0.932	0.003
Population estimate	0.001 (−0.001, 0.003)	0.001	1.240	0.215	0.685
Housing units estimate	−0.001 (−0.002, 0.001)	0.001	−0.800	0.424	−0.099
Households estimate	0.001 (−0.002, 0.004)	0.002	0.693	0.489	0.178
Persons below poverty estimate	0.001 (−0.002, 0.003)	0.001	0.393	0.695	0.087
Civilian (16+) unemployment estimate	<0.001 (−0.008, 0.008)	0.004	0.042	0.967	0.005
Persons (25+) with no high school diploma estimate	−0.003 (−0.007, 0.002)	0.002	−1.156	0.248	−0.320
Persons 65+ estimate	<0.001 (−0.003, 0.003)	0.001	−0.185	0.853	−0.029
Persons 17 and younger estimate	−0.005 (−0.008, −0.001)	0.002	−2.794	0.005	−1.047
Civilian noninstitutionalized population with a disability estimate	0.004 (<0.001, 0.009)	0.002	1.863	0.063	0.299
Single parent household with children under 18 estimate	−0.002 (−0.014, 0.010)	0.006	−0.361	0.718	−0.068
Minority (all persons except white, non-Hispanic) estimate	<0.001 (−0.001, 0.002)	0.001	0.561	0.575	0.181
Persons (age 5+) who speak English “less than well” estimate	0.003 (−0.001, 0.007)	0.002	1.307	0.192	0.282
Housing in structures with 10 or more units estimate	−0.003 (−0.001, 0.007)	0.002	1.316	0.188	0.167
Mobile homes estimate	−0.002 (−0.006, 0.001)	0.002	−1.199	0.231	−0.139
Housing in structures with 10 or more units estimate	−0.002 (−0.015, 0.011)	0.007	−0.311	0.756	−0.057
Households with no vehicle available estimate	0.012 (−0.005, 0.029)	0.009	1.378	0.168	0.211
Persons in group quarters estimate	−0.001 (−0.003, 0.001)	0.001	−1.070	0.285	−0.180
Percentage of persons below poverty estimate	0.012 (−0.128, 0.152)	0.071	0.169	0.866	0.198
Unemployment rate estimate	0.004 (−0.183, 0.192)	0.095	0.044	0.965	0.067
Per capita income estimate	1.510E−4 (0.000072, 0.000214)	<0.001	4.104	0.000	0.419
Percentage of persons with no high school diploma (age 25+) estimate	0.100 (−0.055, 0.255)	0.079	1.270	0.205	1.196
Percentage of persons 65+ estimate	0.057 (−0.076, 0.189)	0.067	0.840	0.401	0.667
Percentage of persons aged 17 and younger estimate	0.211 (0.012, 0.410)	0.101	2.080	0.038	0.350
Percentage of civilian noninstitutionalized population with a disability estimate	−0.169 (−0.368, 0.031)	0.102	−1.659	0.098	−2.747
Percentage of single parent households with children under 18 estimate	0.034 (−0.134, 0.202)	0.086	0.393	0.695	0.050
Percentage minority (all persons except white, non Hispanic) estimate	0.011 (−0.066, 0.087)	0.039	0.274	0.784	0.055
Percentage of persons (age 5+) who speak English “less than well” estimate	−0.096 (−0.324, 0.133)	0.116	−0.823	0.411	−0.149
Percentage of housing in structures with 10 or more units estimate	−0.005 (−0.081, 0.071)	0.039	−0.133	0.894	−0.014
Percentage of mobile homes estimate	0.070 (4.490E−4, 0.139)	0.035	1.976	0.049	1.274
Percentage of occupied housing units with more people than rooms estimate	0.025 (−0.141, 0.190)	0.084	0.294	0.769	0.042
Percentage of households with no vehicle available estimate	−0.130 (−0.373, 0.113)	0.124	−1.052	0.293	−2.347
Percentage of persons in group quarters estimate	0.138 (−0.015, 0.292)	0.079	1.754	0.080	0.263
Sum of series themes	0.098 (−0.205, 0.401)	0.155	0.634	0.526	1.772
Percentage uninsured in the total civilian noninstitutionalized population estimate	0.116 (−0.063, 0.296)	0.091	1.273	0.203	1.891
Estimated daytime population, LandScan 2018	1.430E−4 (0.072E−4, 2.140E−4)	0.036E−4	3.956	0.083E−4	0.156

Although these were the only four factors which exhibited statistical significance at the designated level of p < .01, the values for Percentage of persons aged 17 and younger estimate (B = 0.211 [95 % CI 0.012 to 0.410], t(820) = 2.080, p = .038) and Percentage of mobile homes estimate (B = 0.070 [95 % CI 0.000449 to 0.139], t(820) = 1.976, p = .049), were statistically significant at a level of p < .05, but not statistically significant based on our significance value of p < .01 (Table 3).

Thus, we reject the null hypothesis which states that there is no relionship between overall social vulnerability indices and number of surgeons and fail to reject the alternative hypothesis which proposes that: the number of surgeons in a census tract will be inversely proportional to the census tract's overall social vulnerability indices. Furthermore, we conclude that increased socioeconomic status is associated with a higher number of surgeons in a census tract.

## Discussion

Based on these results, this study reinforces the conclusions made by previous literature, which propose that access to medicine in the United States is unequally distributed along geographic and socioeconomic lines [2]; although previous studies into these disparities primarily focused on the distribution of primary care physicians, the significance of this study lies in its demonstration that the same patterns can be seen in access to surgeons [[4], [5], [6]]. Specifically, this study shows that a higher “per capita income estimate” was significantly associated with a higher number of surgeons in a particular census tract, which provides further insight into the barriers to health experienced by significant portions of the population. The relationships between number of surgeons and “estimated day population,” as well as “Population Estimate” are also in line with previous studies, which describe how surgeons tend to concentrate in high population density urban centers, whether due to resource requirements, social motivations, or monetary incentives [7]. Thus, the relationships^3^ exhibited in this study demonstrate how the continued migration of surgeons to more affluent areas has led to a continuous decline in the accessibility of surgery for those in poverty [2,11,18].

However, some arguments against the linkage between surgical access and socioeconomic status purport a merely coincidental relationship between the two. For example, some suggest that median income increases alongside surgeon number because the income of surgeons artificially inflates the median income of the areas in which they reside; however, other associations, such as the relationship between minority population and surgeons, household composition, and housing type/transportation access, demonstrates that this pattern is more so due to socioeconomic factors, rather than the statistical impact of surgeon incomes on a particular community (Table 1). Others propose that, in spite of these geographic disparities in medical access, there are no “significant differences” in terms of self-perception of health, physical/role functioning, conceding only that there are community-related disparities in “social functioning” [19]. This viewpoint proposes that social health and physical health are independent of one another, but fails to consider that factors like education, food instability, and levels of environmental pollutants have powerful impacts on one's health outcomes, thus leading to generational disparities in overall health and life expectancy in MUAs [20]; this is especially apparent in the higher rates of chronic diseases such as cancer [13].

The IE serves as an insightful demonstration of how this unequal burden of health upon marginalized populations affects health outcomes. A closer examination of Linda University's 2016 Community Health Assessment demonstrates that even within San Bernardino County, a skewed geographic distribution of the social determinants surrounds each city's overall health [12]. For instance, while citizens in Loma Linda are more readily able to access high quality healthcare, due to both higher median income and convenient access to a hospital, members of their neighboring city of San Bernardino, as well as other cities in the IE, struggle significantly more: SVI analysis of San Bernardino in 2018 shows an overall SVI score of 0.9827, indicating very high social vulnerability in all four measured areas: Socioeconomic status, household composition and disability, minority status and language, and housing type and transportation. In contrast, Loma Linda has an overall SVI score of 0.5372, indicating moderate social vulnerability - Loma Linda's social vulnerability is concentrated heavily in minority status and language, and moderately in housing type and transportation, rather than in socioeconomic barriers such as insurance coverage [10]. These differing levels and types of vulnerability have led to proportionally different rates of hospitalization as well as chronic health conditions [12,13].

Although expansion of programs like the Affordable Care Act and Medi-Cal have led to decreases in the numbers of uninsured adults in the United States, the number of individuals who can regularly access medical care still seeks to be improved [21]. Research by DeVoe et al. (2007) demonstrates that the economic and social barriers preventing families from accessing regular medical care extend beyond merely having insurance coverage [22]. For instance, their work indicates that in accessing primary care physicians, families often face a series of various obstacles which include looking for practices that accept public insurance, maintaining all the requirements for keeping Medicaid, and travelling longer distances to meet with physicians who they trust and with whom they feel comfortable. These barriers to healthcare, whether due to lack of local hospitals, inadequate transportation, or overburdening of physicians, heavily impact access to specialties like surgery and exacerbate the already worsening national surgical shortage. In 2004, Solucient, a healthcare consultation firm, released a follow up study to that of The Graduate Medical Education National Advisory Committee in 1980, which recommended that ideal surgical coverage requires “six general surgeons per 100,000 people” [23]. However, the U.S. Census Bureau and the AAMC indicate that in spite of this high demand for surgery, many areas in the United States, particularly rural and impoverished areas, experience a current critical lack of access to surgeons [8]. Thus, our investigation demonstrates, in spite of the tireless work performed by “safety-net hospitals” and social services programs, there continues to be “poor access to surgical services for the urban underserved” [24].

## Limitations and future research

Although our work provides insight into a “model” area for the exploration of surgical inequity across class, race, and social lines, it is not without limitations. For instance, while this data offers powerful insight into the disparities in total surgeon access, different surgical fields require different levels and types of equipment and resources (e.g., general versus transplant versus cardiothoracic). Thus, future research may benefit from exploring the patterns of distribution for surgical subspecialties, providing a more nuanced understanding of the differences in access to, as well as need for, specific surgical care [17].

Furthermore, we recognize that statistically significant results can often be gleaned via the introduction of large sample sizes. However, although some individual relationships (e.g., number of housing units, and number of minorities) indicate only slight patterns of correlation, while simultaneously exhibiting large p values, when examined as a whole, the relationship between SVIs and number of surgeons per census tract exhibits a convincing negative correlation.

Somewhat surprising was the correlation with a smaller “Number of persons aged 17 and younger estimate”.^4^ Future research may benefit from questioning how this variable is impacted by other confounding variables, such as the continued decrease in number of pediatric inpatient units or greater need for surgeries in the non-pediatric population [25,26]. Finally, similarly to previous work, our investigation does “not allow us to […] examine potential differences in quality of care,” which may drastically influence or exacerbate the already existing disparities in access [1].

## Relevance for future practice

An unintended consequence of this study lies in the discovery that the majority of census tracts surveyed within the IE had zero surgeons, thus reinforcing the notion that many communities are without surgeons at all [7,8]. Some potential avenues for beginning to solve this crisis include: increasing resident caps for general surgery, mandating rotations in underserved communities, or incorporating specialized training rotations for surgeons interested in underserved and rural work, so that they may more quickly begin to serve these communities. Thus, this work continues to demonstrate the importance of increasing physician, specifically surgeon, presence in the IE^5^ as well as the greater United States [1,24].

Finally, current work in developing “socially responsible surgery” programs has begun to gain traction, as “a number of dedicated programs at academic medical centers now exist to support scholarship” [24]. This movement not only raises awareness regarding the surgical alleviation of disease burden, but also demonstrates how many surgeons, especially in rural communities, also maintain roles as primary care providers and consistent advocates for their patients [24,27]. Thus, by describing a surgeon's role in not only individual, but also population health, this research hopes to contextualize the work of surgeons within the health needs and characteristics of a community, motivate interested medical students to pursue surgery, and inspire those students and physicians to make positive change in their own lives and communities [8,28].

## Ethics approval

This is an observational study. The Loma Linda University IRB has confirmed that no ethical approval is required.

## Funding sources

This research was supported, in part, by an Alpha Omega Alpha Carolyn L. Kuckein Student Research Fellowship. This funding source was not involved in the study design, the collection, analysis, and interpretation of data, the writing of the report, or the decision to submit the article for publication.

## CRediT authorship contribution statement

**Brandon J. Shin:** Conceptualization, Formal analysis, Funding acquisition, Investigation, Methodology, Resources, Writing – original draft, Writing – review & editing, Supervision. **M. Daniel Wongworawat:** Funding acquisition, Methodology, Writing – review & editing. **Marti F. Baum:** Conceptualization, Funding acquisition, Methodology, Supervision.

## Declaration of competing interest

The authors declare that the research was conducted in the absence of any commercial or financial relationships that could be construed as a potential conflict of interest.

## Footnotes

3 Of note, while the “percentage of mobile homes estimate” fails to meet the specified statistical cutoff of p < .01, its statistical significance at the traditional cutoff of 0.05 advocates for continued consideration and monitoring of its potential impact on a community's access to surgeons (Table 3).

4 Percentage of persons aged 17 and younger estimate is a different means by which to analyze a census tract's sub-17-year-old populus. Thus, its significance at a level of p < .05, but not p < .01, mirrors and reinforces that number of persons aged 17 and younger estimate is correlated of number of surgeons (Table 3).

5 Due to the large geographic area contained within the Inland Empire, a larger number of physicians are required to meet the aforementioned ideal ratio of six surgeons for every 100,000 people [8].
